# Nomogram for subsyndromal delirium after cardiac surgery with cardiopulmonary bypass: a retrospective cohort study

**DOI:** 10.3389/fmed.2026.1837462

**Published:** 2026-06-17

**Authors:** Min Lin, Yuping Xiang, Tianhui Luo, Yan Ren, Jing Wang, Xiaolan Wang, Ling Zeng, Jingxiu Fan

**Affiliations:** 1 Department of Critical Care Medicine, West China Hospital, Sichuan University/West China School of Nursing, Sichuan University, Chengdu, Sichuan, China; 2 Department of Cardiovascular Surgery, West China Hospital, Sichuan University, Chengdu, Sichuan, China

**Keywords:** cardiac surgery, cardiopulmonary bypass, nomogram, retrospective cohort study, subsyndromal delirium

## Abstract

**Objective:**

The incidence of subsyndromal delirium (SSD) after cardiac surgery with cardiopulmonary bypass (CPB) is relatively high. In this study, we aimed to analyze the risk factors associated with postoperative SSD and to construct a predictive model to facilitate early identification of SSD, thereby providing an effective clinical prediction tool.

**Methods:**

We conducted a retrospective cohort study, including patients who underwent cardiac surgery with CPB at a tertiary hospital in Sichuan Province from January 2023 to March 2024. Clinical nurses performed six daily assessments for SSD by using the Confusion Assessment Method for the Intensive Care Unit until delirium occurred or the patient was discharged from the intensive care unit or died. A total of 994 patients were included and divided into a non-SSD group (*n* = 832) and an SSD group (*n* = 162). Univariate and multivariable logistic regression analyses were performed to identify independent risk factors for postoperative SSD. A nomogram was constructed using R software, and the model’s discriminative ability was evaluated using the area under the receiver operating characteristic (ROC) curve.

**Results:**

Among the 994 patients who underwent cardiac surgery, the incidence of SSD was 16.30% (*n* = 162), and 46.30% (*n* = 52) of these patients subsequently progressed to delirium. Multivariable logistic regression analysis showed that age (OR = 1.039), body mass index (OR = 1.078), the use of pain medication after extubation (OR = 2.918), postoperative stress hyperglycemia (OR = 1.848), a postoperative minimum albumin level < 33.4 g/L (OR = 1.97), and a postoperative minimum hemoglobin level < 90 g/L (OR = 2.284) were independent risk factors for SSD after cardiac surgery. A risk prediction model was constructed based on these variables. The area under the ROC curve was 0.744 (95% CI [0.701–0.787]; *p* < 0.001), indicating moderate predictive accuracy of the model.

**Conclusion:**

The incidence of SSD after cardiac surgery with CPB was high. The predictive model developed in this study demonstrated good predictive performance and may assist healthcare professionals in the early identification of high-risk patients, enabling timely preventive interventions to reduce the risk of postoperative SSD.

## Introduction

Subsyndromal delirium (SSD) refers to a condition in which patients exhibit one or several symptoms of delirium but do not meet the full diagnostic criteria for delirium (1). This common condition has an insidious onset and a relatively poor prognosis. A meta-analysis revealed that the postoperative incidence of SSD is approximately 30% (2), and another meta-analysis reported incidence among critically ill patients is 32.4% (3). Patients with SSD experience a decline in cognitive function in the 3 months after hospital admission (4). SSD is also associated with multiple adverse outcomes (5), including an increased risk of mortality (6, 7), prolonged hospital stay (8), and higher likelihood of progression to delirium (9). These consequences negatively affect patients’ quality of life and increase the burden on family caregivers and healthcare systems. Therefore, accurate screening and early prevention of SSD are essential to prevent progression to delirium and to improve patient outcomes.

Cardiac surgery with cardiopulmonary bypass (CPB) is one of the primary treatment approaches for valvular heart disease and coronary artery disease. Globally, approximately two million patients undergo cardiac surgery each year (10). In 2024, 360,000 cardiac surgeries were performed in China, of which over 200,000 involved CPB (11). CPB procedures typically require hypothermic circulatory arrest, which can trigger a systemic inflammatory response, potentially causing neurological injury and postoperative SSD. The reported incidence of SSD following cardiac surgery with CPB ranges from 11.48 to 72.44% (12, 13). Research on SSD after cardiac surgery has primarily been focused on the identification of risk factors (7, 12, 14–16), which include a variety of clinical and perioperative variables. The construction of predictive models that integrate multiple risk factors may enable the early identification of patients at high risk for SSD and support clinical decision-making.

Most SSD prediction models have been tailored for critically ill patients (17–22), and two have been developed specifically for SSD after cardiac surgery (12, 14). Cai et al. (14) included 368 patients undergoing CPB cardiovascular surgery and identified age, the American Society of Anesthesiologists classification, surgery duration, mechanical ventilation time, and platelet transfusion volume as independent risk factors for SSD; their model yielded an area under the receiver operating characteristic (ROC) curve (AUC) of 0.795. Liu et al. (12) included 549 patients who underwent cardiac surgery, some of whom underwent non-CPB procedures, and identified nine predictive factors. However, no risk-specific predictive model exists for SSD after cardiac surgery with CPB.

Our hospital is the largest cardiac surgery center in Southwest China. Building upon the abovementioned studies, we aimed to analyze the risk factors for SSD after cardiac surgery with CPB and develop a predictive model. This model is intended to assist healthcare professionals in the early identification of high-risk patients and improve postoperative outcomes by allowing them to address modifiable risk factors.

## Methods

### Study setting and participants

This retrospective cohort study was conducted in the cardiac surgery intensive care unit (ICU) of a tertiary teaching hospital in Southwest China and was approved by the Ethics Committee of West China Hospital, Sichuan University (approval no.: 2024-848). As this was a retrospective study, the need for written informed consent was waived by the Committee. Adult patients admitted to the ICU after cardiac surgery from January 2023 to March 2024 were consecutively included. Inclusion criteria were as follows: age ≥ 18 years; undergoing cardiac surgery with CPB (valve replacement or repair, coronary artery bypass grafting, or congenital heart defect surgery); and an ICU stay ≥ 24 h. Exclusion criteria were as follows: a history of psychiatric disorders; new-onset postoperative stroke; an inability to undergo delirium assessment during the ICU stay owing to endotracheal intubation or tracheostomy; being in a coma during the ICU stay (Richmond Agitation-Sedation Scale score of −4 to −5); incomplete data; and repeat cardiac surgery.

### Sample size

We estimated the required sample size for predictive modeling by using the following formula (23): *N* = the number of independent variables × (5~10) ÷ the incidence rate. Based on previous studies (12, 14–16), we anticipated that 10 variables would be independently associated with SSD. The reported incidence of SSD after cardiac surgery with CPB ranges from 11.48 to 72.44% (12, 13). Considering that each independent variable requires 5–10 events for sufficient statistical power, and allowing for a 10% rate of invalid or incomplete responses, the required sample size for this study was153-967.

### Variables

The candidate predictive factors were screened based on the following search terms: “Cardiac Surgical Procedures (mesh), Coronary Artery Bypass (mesh), Cardiac Surgery, Cardiovascular surgery, Heart Surgery, Cardiac Surgical Procedure*, Heart Surgical Procedure*, Coronary Artery Bypass Grafting, Coronary Artery Bypass*, Aortocoronary Bypass*, Coronary Artery Bypass Surgery, CABG, myocardial revascularization, Heart Valve Prosthesis Implantation, valve replacement, heart valve replacement surgery, Valve Implantation, Cardiopulmonary bypass” AND “subsyndromal delirium (mesh), sub syndromal delirium, subclinical delirium, subthreshold delirium, subdelirium, acute confusion state, altered mental status”. Search the following databases: PubMed, Embase, Web of Science, proQuest, CINAHL, CENTRAL, CNKI, Wan Fang, Chinese Biology Medicine, Wei pu data, search completed by October 2025. We initially identified 17 candidate predictors (12, 14–16), supplemented by risk factors and predictive model studies of SSD in critically ill patients (3, 17, 18, 22, 24, 25). Based on their reported frequency and the feasibility to obtain these variables in a clinical setting, we ultimately selected 44 candidate predictors for SSD (7 demographic variables, 16 preoperative variables, 8 intraoperative variables, and 13 postoperative variables).

The demographic variables were age, sex, body mass index (BMI), education level, marital status, smoking history, and alcohol use history. The preoperative variables were hypertension, diabetes mellitus, coronary heart disease, cerebral infarction, pneumonia, chronic kidney injury, New York Heart Association (NYHA) class ≥ 3, the hemoglobin level, the white blood cell count, the neutrophil percentage, the albumin level, the blood glucose level, the creatinine level, the triglycerides level, the cholesterol level, and the left ventricular ejection fraction (LVEF).

The intraoperative variables were the surgery duration, the CPB time, the aortic cross-clamp time, the highest intraoperative blood glucose level, the highest intraoperative lactate level, emergency surgery, the surgical approach, and intraoperative blood transfusion. The postoperative variables were sedative use prior to extubation, analgesic use after extubation, the use of pulse index continuous cardiac output for a low cardiac output, blood transfusion, postoperative stress hyperglycemia (PHG), pneumonia, the Acute Physiology and Chronic Health Evaluation (APACHE II) score, the highest lactate level, the highest total bilirubin level, the lowest albumin level, the lowest hemoglobin level, the highest white blood cell count, and the duration of mechanical ventilation. PHG was defined as two random blood glucose measurements ≥ 10.0 mmol/L (180 mg/dL) in the 24 h after surgery.

### Diagnosis of SSD

SSD was assessed using the Confusion Assessment Method for the ICU (CAM-ICU), a validated delirium screening tool suitable for use by non-psychiatric healthcare professionals. It is used to evaluate four features: (1) an acute onset or fluctuating course, (2) inattention, (3) disorganized thinking, and (4) an altered level of consciousness. SSD is defined as the presence of one or more CAM-ICU features without meeting the full criteria for delirium (18). The CAM-ICU has demonstrated a sensitivity of 97.3%, specificity of 96.8%, and Kappa coefficient of 0.895 (26). SSD assessments were performed by trained clinical nurses. From the time that patients awoke after surgery, CAM-ICU evaluations were conducted six times daily (8:00, 12:00, 16:00, 20:00, 0:00, 4:00) and recorded in the electronic medical record system until delirium occurred or the patient was discharged from the ICU or died.

### Data collection

A data collection team comprising six clinical nurses and one critical care specialist was established to gather study variables. An online data collection form was created, and all team members received standardized training to ensure consistency in variables collected, collection timing, and proficiency in navigating the electronic medical record system. All variables were extracted from this system for this study. Preoperative laboratory values and LVEF were based on the final measurements prior to surgery; intraoperative variables were obtained from the surgical and anesthesia records; and postoperative laboratory values were collected from ICU records.

### Statistical analysis

Statistical analyses were performed using SPSS Statistics for Windows version 26.0 (IBM Corporation, Armonk, NY, USA) and R software version 4.1.0 (The R Foundation for Statistical Computing, Vienna, Austria). Quantitative variables with a normal distribution were expressed as means ± standard deviations and compared between groups by using the independent-samples t-test. Quantitative variables that were not normally distributed were presented as medians (percentile 25, percentile 75) and compared using the Mann–Whitney *U* test. Categorical variables were expressed as counts and percentages. Comparisons of unordered categorical variables between groups were conducted using the chi-square test or Fisher’s exact test, while ordered categorical variables were compared using the Wilcoxon rank-sum test.

Variables that were statistically significant upon univariate analysis were included as independent variables in an unconditional binary logistic regression analysis. Independent variables were entered using a forward stepwise approach based on the bias-corrected maximum likelihood estimation. This analysis identified independent risk factors for SSD after cardiac surgery, and a nomogram was constructed to visualize the contribution of each risk factor. The discriminative ability of the model was evaluated using the AUC. Clinically, an AUC > 0.8 is considered to indicate strong diagnostic accuracy, with higher values reflecting better differentiation between high-risk and low-risk patients. A two-sided *p* < 0.05 was considered statistically significant.

## Results

### Patient characteristics

**Figure 1:**
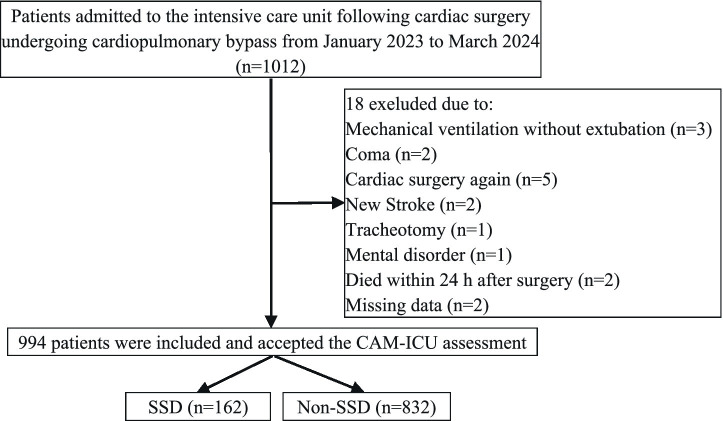
Flow chart of patients in study.

From January 2023 to March 2024, 1,012 patients underwent cardiac surgery with CPB and were subsequently admitted to the ICU. Of these, we excluded 18 patients, leaving 994 for analysis in the study (Figure 1), of whom 162 were diagnosed with SSD, yielding an incidence of 16.30%.

### Univariate analysis

Table 1 summarizes the demographic and preoperative characteristics of the two groups. The mean age of the study population was 57 years, 51.61% were male, and the majority had a lower educational level, with 49.70% having completed secondary school. Hypertension was present in 25.05% of patients, and coronary artery disease in 18.71%; the mean preoperative LVEF was 63%. Univariate analysis revealed significant differences between the SSD and non-SSD groups in term of age, BMI, education level, hypertension, diabetes mellitus, coronary artery disease, a history of stroke, and an NYHA class ≥ 3.

**Table 1:** Preoperative variables of patients in different groups.

Variables	Total (*n* = 994)	non SSD group (*n* = 832)	SSD group (*n* = 162)	*p-*value
Age (years)	57 (50–64)	56 (50–63)	61 (55–68)	<0.001*
Males, *n* (%)	513 (51.61)	421 (50.60)	92 (56.79)	0.149
BMI, kg/m^2^	23.44 (21.18–25.60)	23.28 (21.09–25.48)	24.18 (21.8–26.57)	0.012*
Educational levels				
Primary and lower, *n* (%)	291 (29.27)	229 (27.52)	62 (38.27)	0.005*
Middle school, *n* (%)	494 (49.70)	416 (50.00)	78 (48.15)	
University and higher, *n* (%)	209 (21.03)	187 (22.48)	22 (13.58)	
Married, *n* (%)	893 (89.84)	745 (89.54)	148 (91.36)	0.477
Smoking history, *n* (%)	239 (24.04)	197 (23.68)	42 (25.93)	0.54
Drinking history, *n* (%)	199 (20.02)	167 (20.07)	32 (19.75)	0.926
Hypertension, *n* (%)	249 (25.05)	197 (23.68)	52 (32.10)	0.024*
Diabetes, *n* (%)	108 (10.87)	82 (9.86)	26 (16.05)	0.02*
CAD, *n* (%)	186 (18.71)	141 (16.95)	45 (27.78)	0.001*
Stroke, *n* (%)	57 (5.73)	41 (4.93)	16 (9.88)	0.013*
CKD, *n* (%)	43 (4.33)	35 (4.21)	8 (4.94)	0.675
Pulmonary infection, *n* (%)	108 (10.87)	92 (11.06)	16 (9.88)	0.659
NYHA ≥3, *n* (%)	433 (43.56)	348 (41.83)	85 (52.47)	0.012*
Hemoglobin (g/L)	135 (122–145)	135 (123–146)	133 (118–143)	0.06
WBC (×10^9^/L)	5.97 (4.90–7.16)	5.99 (4.93–7.15)	5.9 (4.83–7.17)	0.806
Neutrophil percentages (%)	59.5 (52.8–66.3)	59.5 (52.8–66)	59.75 (53.1–68.4)	0.594
Albumin (g/L)	42.8 (40–45.5)	42.9 (40.2–45.5)	42.7 (38.7–45.2)	0.122
Blood glucose (mmol/L)	5.06 (4.63–5.82)	5.03 (4.63–5.79)	5.15 (4.65–5.92)	0.198
Creatinine (g/L)	78 (66–90)	77 (66–90)	78 (67–90)	0.542
Triglyceride (mmol/L)	1.23 (0.93–1.67)	1.22 (0.94–1.67)	1.26 (0.9–1.65)	0.742
Cholesterol (mmol/L)	4.24 (3.50–4.94)	4.24 (3.52–4.9)	4.19 (3.36–5.08)	0.84
LVEF (%)	63 (57–69)	63 (58–69)	62 (55–70)	0.356

Median (25th–75th percentiles) for variables without normal distribution; BMI, body mass index; CAD, coronary artery disease; CKD, chronic kidney disease; NYHA, New York Heart Association; WBC, white blood cells; LVEF, left ventricular ejection fraction.

*The statistical significance was set at a *p-*value less than 0.05.

Table 2 presents the intra- and postoperative characteristics. The mean surgery duration was 255 min, CPB time was 126 min, and aortic cross-clamp time was 91 min. Emergency surgery accounted for 7.04% of cases, and valve surgery was the predominant procedure (81.79%). The mean APACHE II score was 16, and the mean duration of mechanical ventilation was 17 h. Univariate analysis revealed significant differences between the SSD and non-SSD groups in terms of the incidence of intraoperative transfusion, incidence of postoperative transfusion, PHG, lowest postoperative albumin level, lowest postoperative hemoglobin level, and duration of mechanical ventilation.

**Table 2:** Operative and postoperative variables of patients in different groups.

Variables	Total (*n* = 994)	non SSD group (*n* = 832)	SSD group (*n* = 162)	*p-*value
Duration of surgery, min	255 (214–302)	254 (213–301)	262 (217–318)	0.083
Duration of CPB, min	126 (95–158)	125 (95–157)	129.5 (96–176)	0.115
Duration of aortic cross-clamp, min	91 (64–119)	90 (64–117)	93 (66–133)	0.126
Maximum intraoperative glucose (mmol/L)	7.1 (6.1–8.5)	7.1 (6.1–8.5)	7.3 (6.2–8.4)	0.396
Maximum intraoperative lactate (mmol/L)	2.9 (2.1–3.9)	2.9 (2.1–4)	2.8 (2.1–3.8)	0.95
Urgent surgery, *n* (%)	70 (7.04)	58 (6.97)	12 (7.41)	0.843
Type of surgery				
Valve replacement, *n* (%)	813 (81.79)	686 (82.45)	127 (78.40)	0.151
CABG, *n* (%)	79 (7.95)	60 (7.21)	19 (11.73)	
Other surgeries, *n* (%)	102 (10.26)	86 (10.34)	16 (9.88)	
Intraoperative blood transfusion, *n* (%)	309 (31.09)	248 (29.81)	61 (37.65)	0.048*
Use sedative medication before extubation, *n* (%)	862 (86.72)	715 (85.94)	147 (90.74)	0.099
Use of pain medication after extubation, *n* (%)	555 (55.84)	447 (53.73)	108 (66.67)	0.002
Low-heart-output use of PICCO, *n* (%)	34 (3.42)	25 (3.00)	9 (5.56)	0.102
Postoperative blood transfusion, *n* (%)	118 (11.87)	89 (10.70)	29 (17.90)	0.009*
Postoperative hyperglycemia, *n* (%)	646 (64.99)	516 (62.02)	130 (80.25)	<0.001*
Postoperative pneumonia, *n* (%)	93 (9.36)	75 (9.01)	18 (11.11)	0.402
APACHE- II socre	16 (15–18)	16 (15–18)	17 (15–18)	0.268
Maximum postoperative lactate (mmol/L)	4 (2.7–6.3)	3.9 (2.6–6.3)	4.15 (2.9–6.4)	0.25
Postoperative highest total bilirubin (μmol/L)	25 (19.1–35.1)	25 (19.1–34.5)	25.85 (18.6–36.8)	0.342
Postoperative minimal albumin<33.4 (g/L)	423 (42.56)	327 (44.71)	96 (59.26)	<0.001*
Postoperative minimum hemoglobin<90 (g/L)	321 (32.29)	239 (28.73)	82 (50.62)	<0.001*
Postoperative highest WBC (×10^9^/L)	12.93 (10.8–15.5)	12.85 (10.87–15.4)	13.36 (10.5–16.1)	0.345
Duration of mechanical ventilation, hour	17 (12–22)	16 (12–21)	19 (14–34)	<0.001*

CPB, cardiopulmonary bypass; CABG, coronary artery bypass grafting; PICCO, pulse indicator continuous cardiac output; APACHE-II, acute physiology and chronic health evaluation-II.

^*^The statistical significance was set at a p value less than 0.05.

### Multivariable logistic regression analysis of SSD

Postoperative SSD was treated as the dependent variable (yes = 1, no = 0) in the multivariable regression model. In total, 14 independent variables were entered. Of these, six were identified as independent risk factors of postoperative SSD. The result shows that, age (OR 1.039; 95% CI [1.02–1.059]; *p* < 0.001), BMI (OR 1.078; 95% CI [1.02–1.14]; *p* = 0.008), use of pain medication after extubation (OR 2.918; 95% CI [1.943–4.381]; *p* < 0.001), PHG (OR 1.848; 95% CI [1.183–2.888]; *p* = 0.007), postoperative minimal albumin<33.4 g/L (OR 1.97; 95% CI [1.363–2.848]; *p* < 0.001), postoperative minimum hemoglobin<90 g/L (OR 2.284; 95% CI [1.556–3.353]; *p* < 0.001) were independent risk factors for SSD after cardiac surgery (Table 3).

**Table 3:** Multivariate logistic regression analysis of SSD in patients with cardiac surgery.

Variables	SE	Wald *χ*^2^ 值	OR	95% CI	*p-*value
Age	0.01	16.148	1.039	1.02–1.059	<0.001
BMI	0.028	7.04	1.078	1.02–1.14	0.008
Use of pain medication after extubation	0.207	26.656	2.918	1.943–4.381	<0.001
Postoperative stress hyperglycemia	0.228	7.276	1.848	1.183–2.888	0.007
Postoperative minimal albumin<33.4 (g/L)	0.188	13.01	1.97	1.363–2.848	<0.001
Postoperative minimum hemoglobin<90 (g/L)	0.196	17.774	2.284	1.556–3.353	<0.001

SE, standard error.

### Nomogram and validation

**Figure 2:**
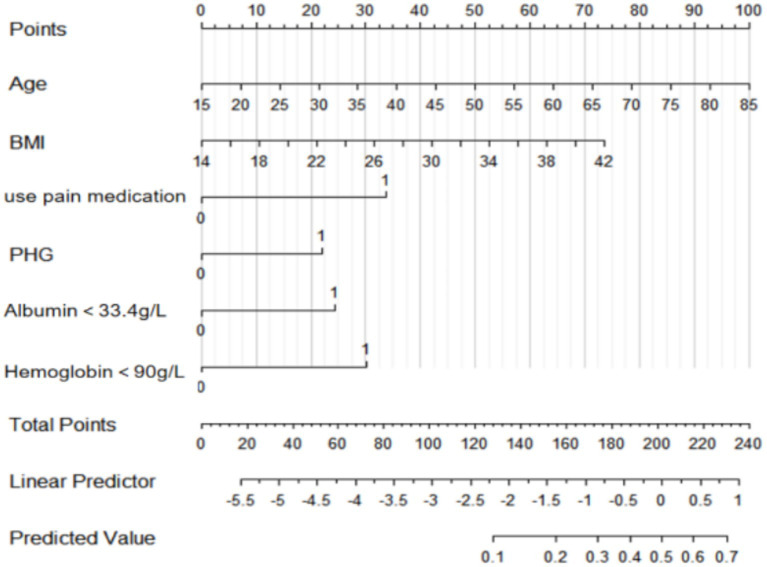
Nomogram of prediction model for SSD in patients with cardiac surgery.

**Figure 3:**
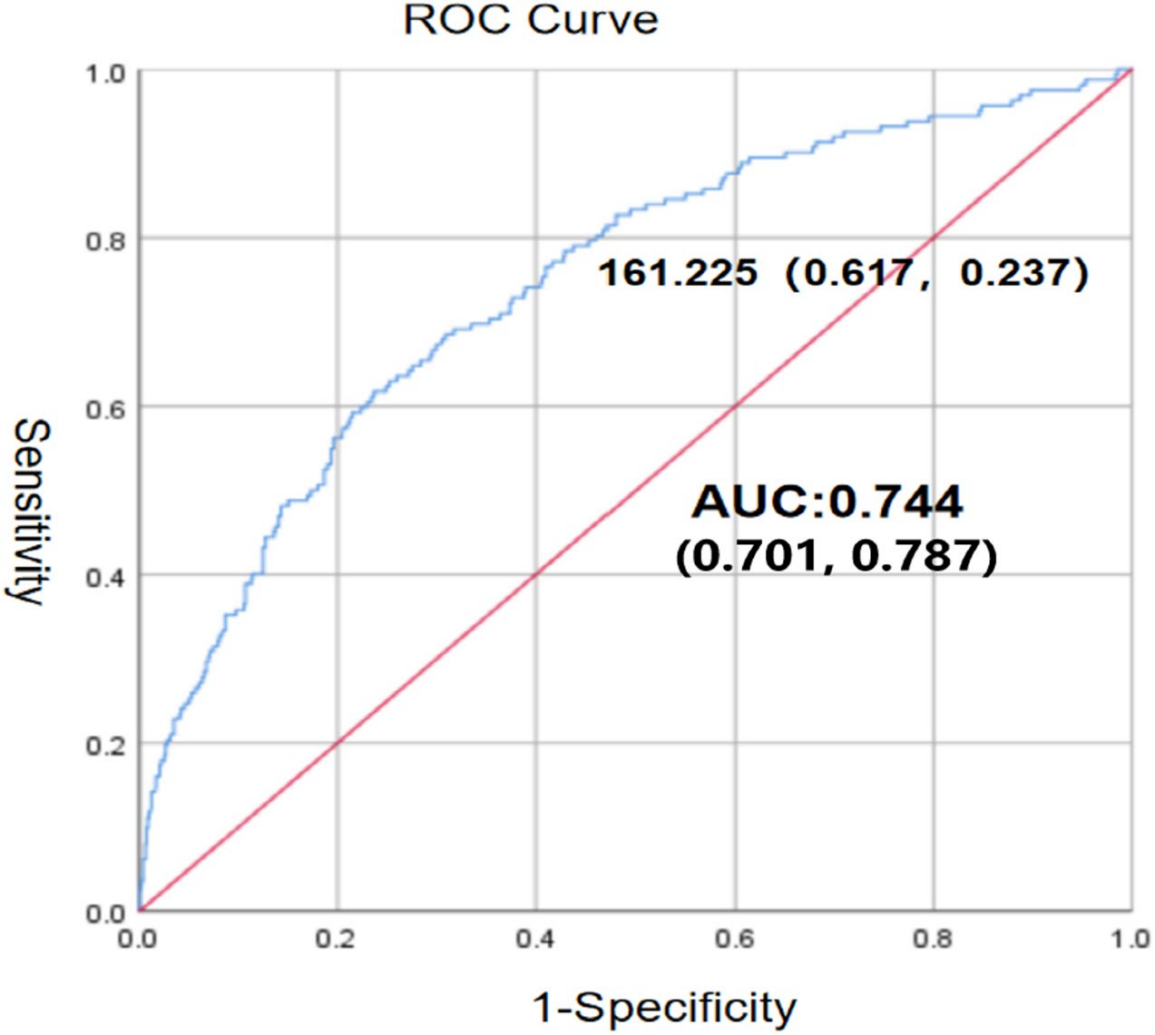
ROC curves of SSD prediction models in a training set for cardiac surgery patients.

The independent risk factors identified in multivariable analysis were incorporated into a predictive model for SSD after cardiac surgery with CPB. Logit (P) = (age-15)*1.43 + (BMI-14)*2.78 + use of pain medication after extubation*39.23 + PHG*22.49 + postoperative minimal albumin<33.4 g/L*24.84 + postoperative minimum hemoglobin<90 g/L*30.26. Accordingly, a nomogram was constructed (Figure 2). In the nomogram, each variable’s value corresponds to a score, and the total score of all variables corresponds to the predicted probability of developing SSD. The predictive performance of the model was evaluated using an ROC curve based on the predicted probabilities (Figure 3). The AUC was 0.744 (95%CI:0.701 ~ 0.787, *p* < 0.001). A cutoff total score of 161 yielded a sensitivity of 61.7% and specificity of 76.3%, indicating that the model has good predictive value.

### Clinical outcome

We analyzed the association between SSD after cardiac surgery and clinical outcomes. We discovered that patients with SSD had significantly longer total hospital stays (14d vs. 12d), longer ICU stays (92 h vs. 67 h), and higher hospitalization costs (14.66 vs. 13.16 ten thousand yuan) than those without SSD. Additionally, the incidence of unplanned ICU readmission was higher in the SSD group. However, the two groups did not significantly differ in terms of rates of self-discharge or in-hospital mortality (see Table 4).

**Table 4:** Postoperative complications in different groups.

Complications	Total (*n* = 994)	Non-SSD group (*n* = 832)	SSD group (*n* = 162)	*p-*value
Length of hospitalization, day	12 (10–17)	12 (10–17)	14 (11–19)	<0.001*
Length of ICU stay, hour	68 (45–97)	67 (44–93)	92 (66–133)	0.001
Unplanned readmission to ICU, *n* (%)	15 (1.51)	9 (1.08)	6 (3.70)	0.024*
Hospitalization costs, 10 thousand yuan	13.35 (9.91–17.07)	13.16 (9.62–16.90)	14.66 (11.62–18.49)	<0.001*
Self-discharge or death, *n* (%)	17 (1.71)	12 (1.44)	5 (3.08)	0.175

Median (25th–75th percentiles) for variables without normal distribution. *The statistical significance was set at a *p* value less than 0.05.

## Discussion

The results of this study indicated that the incidence of SSD after cardiac surgery with CPB was 16.3%, higher than that reported by Liu et al. (12) (11.48%) and Zhou et al. (16) (13.37%), but lower than those in other studies of patients who underwent cardiac surgery (7, 14, 15, 27–29) or were critically ill (2, 3). The low rate in our study may be attributed to the high frequency of SSD assessments in our center, early implementation of sedation and analgesia protocols, and use of multidisciplinary early mobilization strategies. We also found that 46.3% of patients with SSD progressed to delirium. Currently, no established pharmacological treatment for SSD exists. Low-dose haloperidol does not prevent SSD from progressing to delirium and may even exacerbate delirium (30). Clinical guidelines therefore recommend bundled non-pharmacological interventions, such as the ABCDEF approach (31). Accordingly, healthcare professionals should prioritize the early recognition and diagnosis of SSD and focus on minimizing or avoiding modifiable risk factors to prevent progression to delirium.

We identified age, BMI, postoperative analgesic use after extubation, PHG, a low postoperative albumin level, and a low postoperative hemoglobin level as significant predictors of SSD after cardiac surgery. Age is a well-recognized risk factor for SSD (2, 3, 22, 24, 25), likely owing to age-related changes in neuronal morphology and decreased cognitive reserve, which contribute to cognitive decline (32). Previous studies (2, 3) have confirmed that cognitive impairment is a risk factor for SSD, and age has also been associated with the duration of SSD (33) and progression to delirium (34). BMI, which was also identified as a risk factor for SSD, is commonly used to assess obesity and overall health, and a higher BMI can increase the risk of hyperlipidemia, insulin resistance, and cognitive dysfunction (35), which may contribute to the development of SSD. However, Ko et al. (36) reported that overweight and obesity in critically ill patients were not independently associated with delirium in a large study. Moreover, Deng et al. (37) found that BMI was a protective factor for postoperative delirium. In future studies, patients should be stratified according to BMI to verify its association with SSD.

Interestingly, our study is the first to identify PHG as an independent predictor of SSD. In our previous study (38), the incidence of stress hyperglycemia after cardiac surgery was high (65.28%), and such patients had an increased risk of postoperative delirium. Liao et al. (39) reported that stress-induced hyperglycemia significantly increased the risk of delirium in older patients (odds ratio [OR] = 9.13). Patients with diabetes also have a higher risk of SSD than non-diabetic patients (40). Zhang et al. (41) found that perioperative hyperglycemia was associated with new-onset neurocognitive disorders after cardiac surgery. The underlying mechanism may involve a PHG-induced release of proinflammatory cytokines, disruption of blood–brain barrier integrity, and neuroinflammation, ultimately impairing neural function and leading to delirium and SSD (42, 43). These findings underscore the importance of managing PHG to maintain blood glucose within an optimal range and reduce the risk of SSD. Currently, there is no definitive consensus on the optimal perioperative blood glucose range for cardiac surgery patients. Current guidelines recommend maintaining blood glucose below 180 mg/dL after cardiac surgery (44).

Regarding postoperative analgesic use after extubation, our center predominantly uses butorphanol, with minimal use of morphine, fentanyl, or hydromorphone. Previous studies have identified pain as a risk factor for SSD (3, 45), and the use of sedative-analgesic agents such as midazolam (46), remifentanil (20), dexmedetomidine (18), and morphine (19) has also been associated with an increased SSD risk. In future, researchers should further explore the impact of different sedative and analgesic regimens on SSD. Although a previous study (47) confirmed that butorphanol provides effective analgesia for patients undergoing cardiac surgery, healthcare professionals should follow guideline-recommended practices for analgesic use, ensure adequate pain control, and closely monitor pain scores.

Postoperative hypoalbuminemia and anemia were also identified as significant risk factors for SSD in this study. After cardiac surgery with CPB, intraoperative blood loss and fluid shifts often lead to reductions in albumin and hemoglobin levels. In our study, 42.56% of patients had a postoperative minimum albumin level < 33.4 g/L, and 32.29% had a minimum hemoglobin level < 90 g/L. Ma et al. (3) also revealed hypoalbuminemia as a risk factor for SSD (OR = 3.539). Albumin is an important indicator of nutritional status, and malnutrition has been shown to increase the risk of postoperative delirium (48), highlighting the need for comprehensive nutritional assessment and timely intervention among patients who underwent cardiac surgery, particularly those with albumin levels < 33.4 g/L. In terms of the hemoglobin level, Previous research (49) has shown that preoperative anemia is a risk factor for delirium after total knee arthroplasty, and a systematic review (50) revealed that anemia is closely associated with cognitive decline, impaired executive function, and dementia, although the underlying mechanisms remain unclear. In future studies, patients should be stratified according to hemoglobin levels to explore their relationship with SSD. These findings suggest that healthcare providers should closely monitor patients for postoperative albumin and hemoglobin levels and intervene as soon as abnormalities are detected.

### Limitations

This study has several limitations. First, it was a single-center, retrospective, cohort study, and the predictive model has not yet been externally validated, which may limit the generalizability of the findings. Second, SSD was assessed solely using the CAM-ICU. Although it is a reliable tool, it does not evaluate the severity of symptoms. The Intensive Care Delirium Screening Checklist is also widely used for SSD assessment, and the grouping of patients might have differed if we used that tool instead. Third, although candidate predictors were selected using a systematic approach, research on risk factors for SSD after cardiac surgery remains limited. Several potentially relevant variables, based on previous studies in critically ill patients, such as preoperative frailty, baseline cognitive function, the use of physical restraints, antibiotic use, comorbidity scores, and postoperative pain scores were not available and hence not included in the analysis. Fourth, regarding postoperative analgesic use after extubation, our center predominantly uses butorphanol, and very few patients receive morphine. The relationship between butorphanol and SSD requires further investigation. Therefore, large, multicenter, prospective studies with a larger range of potential predictors are needed to validate predictive models internally and externally, and risk factors for progression from SSD to full delirium should also be analyzed.

## Conclusion

The incidence of SSD after cardiac surgery with CPB is high, with a substantial risk of progression to delirium. In this study, a predictive model for SSD after cardiac surgery was developed. Age, BMI, postoperative analgesic use after extubation, PHG, a low postoperative albumin level, and a low postoperative hemoglobin level were identified as independent risk factors, and the model demonstrated good predictive performance. Based on this model, a nomogram was constructed to provide a simple and efficient tool for healthcare professionals to identify patients at high risk of SSD at an early stage. With this predictive model, healthcare professionals can perform risk stratification and implement individualized preventive strategies, thereby reducing the incidence of SSD and the risk of progression to delirium.

## Data Availability

The raw data supporting the conclusions of this article will be made available by the authors, without undue reservation.
